# *ISOLDE*: a physically realistic environment for model building into low-resolution electron-density maps

**DOI:** 10.1107/S2059798318002425

**Published:** 2018-04-11

**Authors:** Tristan Ian Croll

**Affiliations:** a Cambridge Institute for Medical Research, University of Cambridge, Wellcome Trust/MRC Building, Cambridge CB2 0XY, England

**Keywords:** model building, real-space refinement, molecular dynamics, visualization, *ISOLDE*

## Abstract

This paper introduces *ISOLDE*, a new software package designed to provide an intuitive environment for high-fidelity interactive remodelling/refinement of macromolecular models into electron-density maps. *ISOLDE* combines interactive molecular-dynamics flexible fitting with modern molecular-graphics visualization and established structural biology libraries to provide an immersive interface wherein the model constantly acts to maintain physically realistic conformations as the user interacts with it by directly tugging atoms with a mouse or haptic interface or applying/removing restraints. In addition, common validation tasks are accelerated and visualized in real time. Using the recently described 3.8 Å resolution cryo-EM structure of the eukaryotic minichromosome maintenance (MCM) helicase complex as a case study, it is demonstrated how *ISOLDE* can be used alongside other modern refinement tools to avoid common pitfalls of low-resolution modelling and improve the quality of the final model. A detailed analysis of changes between the initial and final model provides a somewhat sobering insight into the dangers of relying on a small number of validation metrics to judge the quality of a low-resolution model.

## Abstract 2

*ISOLDE* is an interactive molecular-dynamics environment for rebuilding models against experimental cryo-EM or crystallographic maps. Analysis of its results reinforces the need for great care when validating models built into low-resolution data.

## 1. Introduction   

As the resolution of a crystallographic or cryo-EM data set degrades, the challenge faced by the model builder increases rapidly as first individual atoms, then small bonded groups and eventually entire residues become effectively unidentifiable from the density alone. The difficulty is further compounded by the fact that low-resolution structures often tend to also be large structures (Supplementary Fig. S1), with thousands or even tens of thousands of residues to contend with. It is unsurprising, then, that the rate of residual errors in published structures similarly grows steeply with decreasing resolution. This fact has long been recognized (Kleywegt & Jones, 1995 ▸), and over the past two decades it has been common to see 3–4 Å resolution structures published with outlier rates 1–2 orders of magnitude higher than would be expected from atomic resolution structures (Croll & Andersen, 2016 ▸). While standards have improved over time (aided in no small part by an ever-increasing supply of high-resolution structures to mine for reference models), it remains common for novel low-resolution structures (with no useful high-resolution homology templates) to be published with statistics indicating high levels of residual error.

### 1.1. Current model-building and refinement tools   

Starting from the state where the practitioner has obtained at least a preliminary map, current methods can be loosely grouped into four partially overlapping categories: automated building of new residues into the map, flexible fitting of an existing structure into a new conformation defined by the map, local refinement and manual inspection/rebuilding. A pioneering (and still current) example of the former is *ARP*/*wARP* (Langer *et al.*, 2008 ▸), which is available as a standalone package or *via* the *CCP*4 suite (Winn *et al.*, 2011 ▸). More recent packages include *phenix.autobuild* (Terwilliger *et al.*, 2008 ▸), which is distributed with the *PHENIX* suite (Adams *et al.*, 2010 ▸), and *Buccaneer* (Cowtan, 2006 ▸), which is available *via* *CCP*4. These packages are often capable of building >90% of residues into maps with a resolution of 3 Å or better, with success rates decreasing rapidly beyond this threshold (Terwilliger *et al.*, 2008 ▸; Cowtan, 2006 ▸; Langer *et al.*, 2008 ▸).

The concept of applying molecular dynamics (MD) to the task of crystallographic structure refinement dates back almost three decades to the release of *X-PLOR* (Brünger, 1992 ▸). In more recent times, MD flexible fitting or MDFF (Trabuco *et al.*, 2008 ▸) was introduced to refit atomic models into low-resolution cryo-EM density by reimagining the electron-density map as a potential energy field causing atoms in a running MD simulation to be attracted into local density maxima. Two notable recent extensions of this concept are *Flex-EM* (Joseph *et al.*, 2016 ▸) and *CryoFIT* (Kirmizialtin *et al.*, 2015 ▸), which each use more complex energy functions based upon cross-correlation between experimental and simulated density maps to reduce the tendency for trapping in local minima. While such methods can be extremely powerful in their ability to ‘morph’ structures from one conformation to another, they tend to implicitly assume that the starting model is essentially ‘perfect’: that is, that any residual errors can be corrected by sufficient MD equilibration and/or annealing. Unfortunately this is by no means guaranteed, particularly in the somewhat common case where the starting model was itself built into a low-resolution map, where it is common to find errors that are sufficiently large that unaided correction is intractable on any currently achievable MD timeframe. An obvious example is cases where portions of the model have been built out of register by one or more residues (Brändén & Jones, 1990 ▸).

Local refinement methods such as *REFMAC* (Murshudov *et al.*, 2011 ▸), *phenix.refine* and *phenix.real_space_refine* (Afonine *et al.*, 2012 ▸) perform far more local searches that aim to simultaneously optimize stereochemical restraints and fit to the data. Such methods by their nature are prone to trapping in local minima, and are explicitly designed to be iterated with (re-)building methods.

Arguably the state of the art in automated model rebuilding is *ROSETTA* (Wang *et al.*, 2016 ▸), which uses extensive, massively parallel fragment-based search methods to optimize a structure, often achieving impressive results even in low-resolution maps. This comes at a substantial computational cost, requiring for example approximately 5000 CPU hours to re-refine a 2392-residue cryo-EM structure, as described in the above reference.

Despite the various successes and continued improvement of the above and similar methods, it remains true now (and arguably for the foreseeable future) that human inspection and, where necessary, adjustment of the model fit to the data is an indispensable part of the model-building workflow. It is thus vital to ensure that the necessary tools improve and evolve alongside automated methods to improve not only the quality of the final outcome, but also the speed and ease with which that outcome is achieved. Recently, I demonstrated the use of interactive MDFF simulations to improve models built into challenging low-resolution crystallographic maps (Croll *et al.*, 2016 ▸; Croll & Andersen, 2016 ▸; Focht *et al.*, 2017 ▸). Here, I introduce *ISOLDE* (Interactive Structure Optimization by Local Direct Exploration), an entirely rebuilt, extensible and scriptable interactive MDFF environment designed for intuitive and high-fidelity remodelling in low-resolution crystallo­graphic or cryo-EM maps. As an illustrative example, I demonstrate its use in rebuilding an existing large 3.8 Å resolution cryo-EM model, and note some surprising observations in the comparison between the starting and final models.

## 2. Overview of the *ISOLDE* design philosophy   

### 2.1. Support for nonspecialist users   

While improving the standards of structures produced into the future is of course an important goal in itself, it is also very important to consider the ever-growing existing structural resource in the wwPDB (134 656 entries at the time that this article was written) in the context of end users. These structures are regularly used by non-expert structural biologists in MD simulations, computational docking/drug design, homology modelling *etc.*, and are typically used ‘as is’ with the expectation that they are essentially correct. While the problem of experimental structures being treated as ‘gospel’ by many end users was noted in the literature almost three decades ago (Brändén & Jones, 1990 ▸), it is concerning that even now many homology-modelling pipelines make no attempt to assess or rank potential templates by quality or resolution.

Substantial effort by others has already gone into addressing this issue, and the recent decision by the wwPDB to allow depositors to submit updated versions of existing structures (http://www.wwpdb.org/news/news) is a promising development. In particular, the *PDB-REDO* server (Joosten *et al.*, 2014 ▸) implements an automated pipeline of optimized refinement and local rebuilding (peptide-bond flips and rotamer adjustments) which can often significantly improve upon deposited models. *PDB-REDO* will not attempt rebuilding of data sets with resolutions beyond 3.5 Å or with less than 2.5 reflections per atom, and does not attempt to diagnose or fix larger-scale errors such as register shifts. While it often yields impressive improvements in, for example, the Ramachandran plot, particularly for models in the 2–3.3 Å resolution range, beyond about 2.5 Å resolution the typical quality of the final model remains well below that expected at higher resolutions (Joosten *et al.*, 2012 ▸). Thus, *PDB-REDO* alone cannot currently fully substitute for human-guided rebuilding, and like other refinement tools is at its most powerful when used in between rounds of human inspection and manual rebuilding.

My goal in the development of *ISOLDE* is therefore twofold: not only should it be powerful enough to act as a workhorse tool for dedicated structural biologists, but it should also provide a gentle learning curve to allow a non-expert to open and inspect a structure and its map(s), and quickly learn how to make meaningful corrections where necessary.

A corollary to this design criterion arises from the fact that many of the target users will not have ready access to high-performance computing resources. Therefore, it is important to ensure that *ISOLDE* can be run usefully on readily available consumer hardware: for example a laptop or desktop computer with one OpenCL or CUDA-capable graphical processing unit (GPU) and at least 8 GB of RAM.

### 2.2. High-quality visualization   

The field of structural biology has its roots in an era when computing power, and in particular graphical rendering ability, was limited and expensive. Software designers were therefore forced to make compromises between rendering quality and speed, and so packages designed for publication-quality rendering were generally separated from the task of model building, where simple, fast line graphics dominated. Thanks in large part to the consumer graphics industry, this separation is no longer necessary: fully rendered scenes of hundreds of thousands to millions of triangles can be readily animated at >30 frames per second on low-cost hardware. The quality of visualization becomes particularly important at low resolution: whereas at high resolution the source of a problem and its solution are usually contained within the space of 1–3 residues, identifying the root cause of an error in a low-resolution structure may require a careful inspection of many dozens of residues at once. It is therefore very important to provide methods to quickly isolate and visualize in a meaningful way any arbitrary selection of residues.

### 2.3. Physically realistic environment   

Interactive modelling environments have historically also been limited in their handling of atomic interactions, in large part owing to the same computational constraints that applied to graphical rendering. Typically only bonded interactions (bond lengths, angles and dihedrals) are considered, while the far more computationally demanding van der Waals and electrostatic interactions are excluded. Given high-resolution data this is often sufficient to yield an excellent result, but as resolution degrades it can lead to an increasingly frustrating and baffling task as atoms slide through each other into physically impossible configurations. The explicit inclusion of nonbonded interactions avoids this issue and makes the model-building experience somewhat more akin to working with a real-world physical model, particularly when combined with the use of a suitable three-dimensional haptic interface, the advantages of which have been discussed in previous work (Croll *et al.*, 2016 ▸; Croll & Andersen, 2016 ▸).

### 2.4. Real-time validation   

One great boon from the rapid growth of the wwPDB has been the ability to mine the subset of atomic resolution structures for statistical information about the real-world distribution of biomolecule conformations. From the extensive early work by various pioneers (Hooft *et al.*, 1996 ▸; Kleywegt, 2000 ▸; Laskowski *et al.*, 1998 ▸) has grown near-comprehensive resources such as *MolProbity* (Chen *et al.*, 2010 ▸; Hintze *et al.*, 2016 ▸) and the wwPDB validation pipeline (Read *et al.*, 2011 ▸), providing very well defined probability distributions, for example, for protein and nucleic acid backbone and side-chain conformations. These are extremely valuable tools, but when (as is somewhat common in a low-resolution structure) the flagged problems in a comprehensive validation plot/table number in the hundreds or even thousands, the task of working through them all can appear insurmountable. With *ISOLDE* I aim to provide fast implementations of these validation metrics, allowing them to be calculated in real time and mapped directly on to the molecule visualization, providing immediate feedback on the results of manipulations without the need to stop and check a separate report.

### 2.5. Scriptability   

While the primary focus of my development is currently on user interaction, the MDFF environment clearly provides many opportunities for the automation of key tasks. To facilitate future development, all of the unit operations described below (including model manipulation, validation and checkpointing) are accessible *via* a Python API.

## 3. Implementation and workflow   

*ISOLDE* is implemented as a Python 3.6 plugin to *UCSF ChimeraX* (Goddard *et al.*, 2017 ▸) and can be installed on Linux and Mac operating systems *via* its ToolShed (Tools/More Tools in the *ChimeraX* menu). Handling of reciprocal-space data and crystallographic symmetry is provided *via* a *ChimeraX* plugin to Clipper-Python (McNicholas *et al.*, 2017 ▸). MD calculations are handled by *OpenMM* 7.1 (Eastman *et al.*, 2017 ▸) using the AMBER ff14sb force field (Maier *et al.*, 2015 ▸) in GB-Neck2 implicit solvent (Nguyen *et al.*, 2013 ▸) with grid-based protein backbone corrections (Perez *et al.*, 2015 ▸). Preliminary support for three-dimensional haptic interaction *via* the CHAI3D library (Conti *et al.*, 2003 ▸) is available on request. While a CPU-only implementation is provided, in practice an OpenCL- or CUDA-capable GPU (with all necessary drivers correctly installed) is required for adequate performance. Illustrative benchmarks for two machines with very different capabilities (a MacBook Air using its onboard GPU and a desktop-replacement gaming laptop with a NVIDIA GTX1070 GPU) are provided in the Supporting Information (§S3). The former supports somewhat interactive simulations up to a few thousand atoms (sufficient for small-scale local remodelling tasks) and is capable of non-interactive settling of the entire 60 000-atom MCM-2 complex. The latter allows interactive speeds up to about 20 000 atoms (on the order of 1000 protein residues).

### 3.1. Preparing for a simulation   

The minimal input for *ISOLDE* is a macromolecular structure consisting of a protein and/or nucleic acid with all atoms (including H atoms) present. Small-molecule ligands other than water and metal ions are not yet supported, and metal ions should be treated with care (as will be discussed further below). It is allowable (and in fact preferable) for the termini of partially built chains to have a missing bond [*i.e.* N-termini built as —C(O)—NH and C-termini as —C^α^(*R*)—CO] unless it is expected that they are true terminal residues. While neither *ChimeraX* nor *ISOLDE* currently include tools for building in missing heavy atoms, H atoms can be con­veniently added using the AddH command in *ChimeraX*. Secondary structure may be recalculated at any time using the *ChimeraX* dssp command.

While it is possible to run interactive simulations in the absence of a map, in most cases at least one electron-density map is desirable. While in future I plan to provide a unified environment with a similar interface for real-space maps and those generated from structure factors, currently only the latter are supported. Real-space maps may be converted *via* the use of *phenix.map_to_structure_factors* (Afonine *et al.*, 2012 ▸). Multiple maps (for example cryo-EM maps with different sharpening parameters, or crystallo­graphic 2*F*_o_ – *F*_c_ and *F*_o_ – *F*_c_ maps) should be bundled into a single MTZ file.

*ISOLDE* starts by default in ‘Map Fitting mode’, which should be used for all tasks involving maps of crystallographic or cryo-EM origin. ‘Free mode’ allows simulation in the absence of any map. With the starting model loaded in *ChimeraX*, the user should click ‘Initialize model/MTZ combo’, make sure that the correct model appears in the ‘Atomic model’ drop-down box, and then click the ‘Map MTZ’ button and choose an MTZ file containing at least one set of amplitudes and phases. Finally, clicking ‘Initialize’ will load and associate the map with the model and provide a visualization involving a scrolling sphere of density similar to that provided by *Coot* (Emsley & Cowtan, 2004 ▸) or *CCP*4*mg* (McNicholas *et al.*, 2011 ▸).

If the model is correctly prepared as described above, starting a simulation involves selecting one or more atoms in the main *ChimeraX* window and then clicking ‘Go’ in the *ISOLDE* window. The selection will be automatically expanded along the chain (by default by three residues in each direction) and further to include all residues with atoms within 5 Å of this selection. These become mobile in the MD simulation, while a further shell of surrounding residues is fixed in space to ensure that the simulation remains in context with its surroundings. When first working with a new structure it is advisable to initially select the entire structure for simulation to relieve any bad clashes, which may otherwise yield destabilizing forces. At any time during the simulation it is possible to store a checkpoint (a snapshot of atomic positions and all custom restraints) which may be returned to (discarding all subsequent changes) at any time. Saving a checkpoint *via* the GUI overwrites any previous one, but any number of checkpoints may be stored *via* the scripting interface. To end a simulation the user may choose to keep the current state, revert to the last checkpoint or discard all changes and revert to the initial state. Since a checkpoint is a snapshot of the running simulation, all stored checkpoints become invalid once the simulation is terminated.

### 3.2. Low-resolution visualization options   

For any structure, it is generally advisable that human eyes should assess every residue against the density at least once (Wlodawer *et al.*, 2013 ▸); quite apart from the need to reduce errors, this increases the chances of identifying unusual sites of potential biological interest. This becomes ever more important as reducing resolution leads to an increased probability of gross modelling errors. Diagnosis of errors in register in particular can become problematic at low resolution when using the commonly used ‘sphere-of-density’ view. The essential problem here is that the error may encompass many dozens of residues along the length of the chain, such that a sphere large enough to cover the whole problem may encompass a vastly larger amount of information irrelevant to the task of diagnosis and correction. A useful alternative visualization approach involves masking the map to within a reasonable distance from a given selection of arbitrary shape (for example a β-hairpin and its immediate surrounds): large enough to provide local context, but small enough to cut out most extraneous detail. This is performed automatically upon starting a simulation, with the mask updated periodically to account for atom movements. Additionally, menu options are provided to mask to any arbitrary selection while viewing the static model, and an additional tool allows the user to ‘step through’ the structure in overlapping steps, each covering two defined secondary-structure elements and any flanking unstructured residues.

### 3.3. Real-time validation   

**Figure 1:**
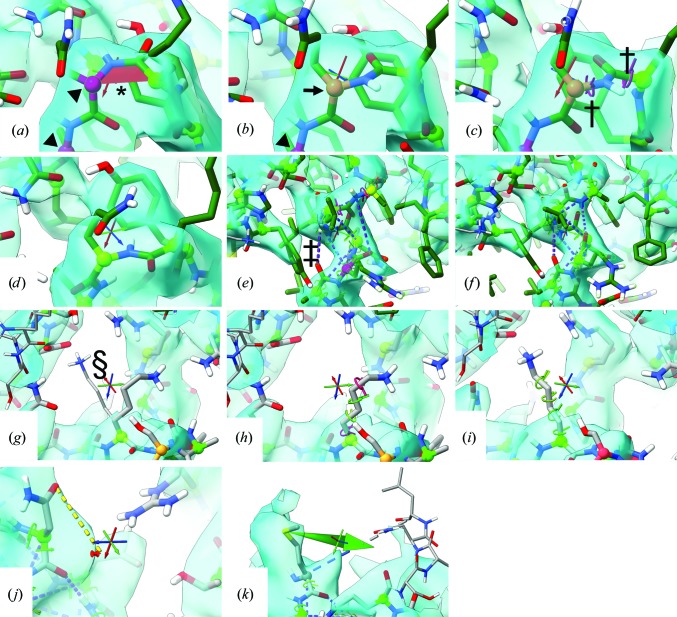
Common annotations and unit operations used during *ISOLDE* simulations. (*a*, *b*) *Cis* peptide bonds (marked with asterisks) are filled with a red trapezoid, while twisted peptide bonds (not shown) are filled in yellow. C^α^ atoms are coloured according to their current Ramachandran status, with outliers (arrowheads) appearing in maroon, marginal conformations shaded from maroon to yellow and preferred conformations shaded from yellow to green with increasing probability. Scripted *cis*–*trans* and peptide-plane flips act on the peptide bond N-terminal to the selected residue. (*c*, *d*) Flipping a peptide plane involves imposing temporary restraints on the φ and ψ dihedrals. Dihedral restraints are annotated by a ring-and-posts motif around each axial bond (marked with daggers), where the angle between the posts gives the current deviation from the target and the colour denotes the level of satisfaction of the restraint. (*e*, *f*) Secondary-structure restraints combine φ and ψ restraints, with distance restraints between O_*n*_ and N_*n*+4_ and between C^α^_*n*_ and C^α^_*n*+2_ displayed as purple dotted pseudobonds (marked with double daggers). (*g*, *h*, *i*) Previews of rotamer options (marked with section symbols) are shown in a thinner stick representation and cycle in order of probability for the given secondary structure. The chosen rotamer coordinates may be committed directly, but it is generally preferable to instead apply the target as dihedral restraints, allowing the atoms to approach the target conformation smoothly without risking clashes. Any heavy atom may be restrained to a given location with a user-defined spring constant (*j*) and/or tugged directly with the mouse or a three-dimensional input device (*k*). All panels are screen captures taken from the *ISOLDE* environment during rebuilding of the MCM2-7 model.

Real-time validation and visualization of protein backbone geometry is implemented as shown in Figs. 1 ▸(*a*) and 1 ▸(*b*). Peptide bonds in the *cis* conformation are highlighted by filling the C^α^—C—N—C^α^ dihedral with a red trapezoid, similar to their representations in *Coot* (Emsley & Cowtan, 2004 ▸) and *KiNG* (Chen *et al.*, 2009 ▸); twisted peptide bonds (not shown) are similarly filled in yellow. An interactive list of all *cis* and twisted peptides in the structure is available through the *ISOLDE* interface. The status of each mobile protein residue on the Ramachandran plot is mapped to the colour of its C^α^ atom, varying as the log of its probability from maroon (*P* < 0.05%; arrowheads) through yellow (0.05% ≤ *P* < 2%; arrows) to green (*P* ≥ 2%). Ramachandran scores are updated every ten simulation frames (approximately once per second under typical use conditions). Note that this annotation provides visual feedback only: no artificial Ramachandran restraints are applied beyond the normal tendency of the MD simulation to settle towards favoured conformations.

### 3.4. Restraints and interactive manipulations   

Most manipulations of the model in *ISOLDE* are achieved *via* the addition and removal of dihedral, position and/or distance restraints to smoothly guide atoms into new configurations. This allows the surrounding atoms to move as necessary to accommodate each change, automatically excluding physically impossible clashes. Upon loading a model, only peptide-bond geometry is restrained by default; a necessity given that the large forces involved in interactive MD can otherwise easily cause inadvertent *trans*–*cis* flips.

It is important to remember that from the perspective of *ISOLDE* there is no difference between a restraint and a target. All of the restraints described below may be used equally to restrain the model to a given starting configuration or to steer large conformational changes.

#### 3.4.1. Dihedral restraints   

Given that MD parameterizations are already tuned to match observed equilibrium di­hedral distributions as closely as possible, it is important to ensure that any restraints allow a reasonable range of motion around any given equilibrium angle. For example, analysis of structures obtained at ultrahigh resolution has found that peptide bonds can regularly twist 10–20° from planar, and that twists of over 30° are possible (albeit extremely rare; Brereton & Karplus, 2016 ▸). Dihedrals are therefore restrained *via* a flat-bottomed potential,
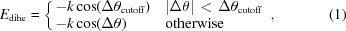
where *E*_dihe_ is the restraint energy, Δθ is the difference between the dihedral angle and target and *k* is a proportionality constant defining the strength of the restraint. By default Δθ_cutoff_ is set to 30° for backbone φ, ψ and ω dihedrals and 15,° for all side-chain χ dihedrals. I chose the cosine instead of the simpler harmonic potential *E*_dihe_ = *k*Δθ^2^ to improve the stability of results when the target is 180° away from the current angle, since the latter form leads to a discontinuity in the energy gradient here leading to unstable behaviour. While use of the cosine potential in principle creates a potentially problematic metastable state (d*E*/dθ = 0) when Δθ = 180°, in practice this is negligible for useful values of *k*.

Flipping a peptide bond from *cis* to *trans* (Figs. 1 ▸ *a* and 1 ▸ *b*) is thus achieved programmatically by changing the value of the associated ω dihedral restraint from 0 to 180°, while flipping the peptide plane (Figs. 1 ▸ *c* and 1 ▸ *d*) involves adding temporary dihedral restraints to φ and ψ, with targets 180° away from their starting values. These restraints are automatically removed once the targets are satisfied, or with a printed warning if the targets are not met within a defined number of steps. For all dihedrals other than ω, an active dihedral restraint is displayed *via* a ‘ring-and-posts’ motif surrounding the axial bond, indicating the current distance from the target by colour and by the angle between the posts. Dihedral restraints are also combined to restrain secondary structures (Figs. 1 ▸ *e* and 1 ▸ *f*) and rotamers (Figs. 1 ▸ *g*, 1 ▸ *h* and 1 ▸ *i*).

#### 3.4.2. Distance and position restraints   

Distance restraints (between a pair of atoms) and position restraints [between an atom and a defined (*x*, *y*, *z*) position] are conceptually very similar. When close to the target, each is defined as a simple harmonic spring, switching to a constant force at larger distances to avoid destabilization of the simulation,
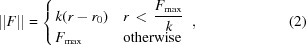
where *F* is the magnitude of the applied force, *k* is the spring constant, *r* is the distance between bonded atoms (or the atom and target positions), *r*_0_ is the target distance (zero for position restraints) and *F*_max_ is the maximum allowed force. At present, distance restraints are only used in secondary-structure restraints (Figs. 1 ▸ *e* and 1 ▸ *f*), but will be extended in future to support metal-ion coordination sites and other situations where classical MD parameterization is insufficient.

Position restraints may be applied to any heavy atom with per-atom user-defined spring constants, and are visualized as a dotted pseudobond connecting the restrained atom to a pin (Fig. 1 ▸ *j*).

#### 3.4.3. Direct tugging   

While the use of interactive restraints often allows fast and/or precise rearrangement, in some cases it is preferable to simply ‘tug’ a given atom into place. Any heavy atom may be tugged using the mouse (Alt + right click/drag) or using a three-dimensional haptic interface if available. In either case, tugging is achieved *via* the application of a moving position restraint of the same form as described above and visualized as a green arrow that rotates and scales according to the direction and magnitude of the tugging force (Fig. 1 ▸ *k*).

## 4. Case study: rebuilding the 3.8 Å resolution MCM2-7 complex   

The recently published 3787-residue yeast MCM2-7 heterohexamer (PDB entry 3ja8; Li, Zhai *et al.*, 2015 ▸) was built into 3.8 Å resolution cryo-EM density starting from homology models generated from a distantly related archaeal homohexamer using *CHAINSAW* (Stein, 2008 ▸), followed by extensive iterations of manual rebuilding in *Coot* and refinement with *phenix.real_space_refine* using Ramachandran and rotamer restraints. Particularly given the scale of the challenge, a cursory glance at the validation statistics provided on any of the PDB webservers suggested no serious cause for alarm: while the clashscore of 28 is certainly high, the numbers of Ramachandran outliers (1.1%) and in particular side-chain outliers (0.1%) are very low for a structure of this resolution. Closer inspection, however, revealed a somewhat more problematic reality.

### 4.1. Model preparation and general refinement strategy   

Since *ISOLDE* is currently unable to handle ligands, the six ADP molecules that are present were removed for simulation purposes and replaced before each refinement in *phenix.real_space_refine* (version dev-2947). Extra care was taken during simulations to prevent adjacent residues from migrating into the vacated density. Missing side chains were added using *Coot*, with no attempt at this stage to optimize geometry other than avoiding bad clashes. In later rounds, zinc ions associated with the five zinc-finger domains and one lysine residue (described below) were placed using *Coot*. H atoms were added using the AddH command in *ChimeraX*. The deposited electron-density map was converted to structure factors and the sharpening *B*-factor was optimized using *phenix.auto_sharpen* (Adams *et al.*, 2010 ▸).

### 4.2. Initial settling and analysis   

**Figure 2:**
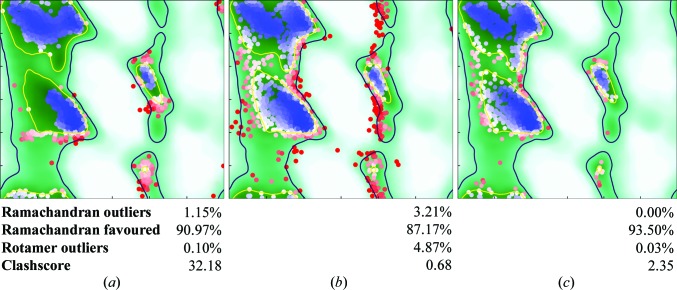
Ramachandran plots for general residues at key stages of rebuilding/refinement. (*a*) PDB entry 3ja8 was originally refined from a largely hand-built model with the aid of Ramachandran and rotamer restraints, achieving a *MolProbity* score of 2.51. Such restraints can be problematic when the nearest allowed region of the plot is not the true conformation for a given residue. (*b*) After energy minimization and 3000 MD steps in *ISOLDE* the number of outliers had increased substantially, suggesting that portions of the original model were indeed overfitted into energetically unfavourable conformations. (*c*) Extensive remodelling and restrained refinement yielded a final model with no Ramachandran outliers and an overall *MolProbity* score of 1.44.

As mentioned above, when working on a new model in *ISOLDE* it is almost always advisable to first run a short, non-interactive simulation of the entire structure in order to resolve bad clashes and other unphysical, high-energy features. In this case I was concerned to note an immediate and significant degradation in the Ramachandran plot (Figs. 2 ▸ *a* and 2 ▸ *b*), with outliers increasing by approximately 2% and favoured residues decreasing by almost 4%. In my experience this is highly unusual: in almost all other cases I have found that settling in the MDFF environment tends to yield an immediate improvement in the Ramachandran plot. Most of the newly created outliers originated from an unusually large clustering of residues in the marginally allowed regions around φ = 30°, suggesting that the use of Ramachandran restraints in the original model had led to force-fitting of many residues into ‘allowed’ but incorrect regions of (φ, ψ) space. A further indication of potentially serious problems was found in the presence of 116 (3.2%) nonproline *cis* peptide bonds, which is a rather common affliction of structures from this era (Croll, 2015 ▸), and a further 19 severely twisted peptide bonds.

Since nonproline *cis* bonds are in reality vanishingly rare, at approximately five in 10 000 residues (Stewart *et al.*, 1990 ▸; Croll, 2015 ▸), tend to be strongly stabilized and well resolved when they do occur, and owing to their unusual conformation may reduce the visibility of other errors, it is sensible to check and where necessary correct these as early as possible. The ‘Peptide bond geometry’ widget on the ‘Validate’ tab of *ISOLDE* can aid in this process. Clicking on any entry in its list of *cis* or twisted peptide bonds selects the associated residue and focuses on it in the main view. Clicking ‘Go’ will then start a localized simulation as described in §[Sec sec3.1]3.1. In most cases the extent of this simulation is sufficient to check and, where necessary, correct the issue by selecting an atom from the residue C-terminal to the *cis* bond, and clicking the ‘*cis* <-> *trans*’ button on the ‘Rebuild’ tab. In each case it is advisable to carefully check the local density for evidence of systematic problems (for example register errors) extending beyond the boundary. If such a problem is suspected, it is best to stop the simulation and restart with a larger initial selection. In this case, none of the *cis* nonprolines nor the five *cis* prolines found in the structure appeared to be justifiable. Many cases appeared in flexible loop or random-coil regions where a *cis* bond is unsupportable (owing to the aforementioned need for strong stabilization of the strained *cis* conformation), while for those appearing in more rigid/well resolved regions modelling in *trans* led to clear improvements in density fit, hydrogen bonding and/or Ramachandran statistics.

### 4.3. Correction of large-scale errors   

**Figure 3:**
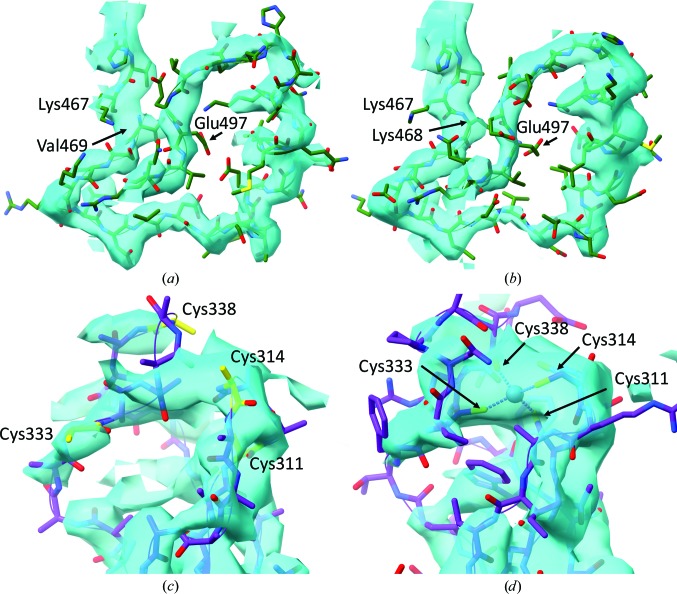
Examples of regions where bulk remodelling was necessary. (*a*) Presumably owing to an erroneous sequence alignment to the homology template, Lys468 of chain 4 was missing, with the following 27 residues shifted in register by one residue to fill the gap. This was corrected using *ISOLDE*’s register-shift function prior to adding the missing residue in *Coot*. (*b*) Final conformation after refinement against the autosharpened map. (*c*) The zinc-finger domain in chain 6 is found in weak and fragmented density, and was originally modelled as a polyalanine trace with the cysteine residues out of position. (*d*) Autosharpening of the map significantly improved interpretability in this region, allowing hand-modelling of this domain into the canonical fold with the coordinated zinc ion present in the centre of the highest density peak.

At this stage it is advisable to step through the structure using the tools described in §[Sec sec3.2]3.2, diagnosing and correcting any gross errors (for example rotamers/segments that are obviously out of density, register errors *etc.*). Until this inspection is complete it is generally not productive to focus on correcting every small issue, since it is possible that your work will be undone by some later major adjustment nearby. While stepping through the structure in this manner I identified one clear register error in chain 4, presumably arising from a misalignment between the sequence and the homology template (Figs. 3 ▸ *a* and 3 ▸ *b*). Here the sequence jumped from Lys467 to Val469 in the middle of well defined density, with no room available for the intervening residue. The structure and fit N-terminal to the break appeared to be reasonable, whereas the following stretch of approximately 30 residues revealed various examples of poor fit to the map and somewhat questionable contacts.

Before attempting a large remodelling task such as this one it is advisable to record a checkpoint, since in many cases finding the correct solution may require testing and discarding a range of hypotheses. Shifts in register are aided by the ‘Register Shift’ widget found on *ISOLDE*’s ‘Rebuild’ tab. The user must select a continuous stretch of protein (which may be grown/shrunk at either end using the tools provided with the widget) and the number of residues to shift by (where a negative number indicates a shift towards the N-terminus). All existing restraints on the selected sequence are removed and three-dimensional parametric splines are fitted to the (*x*, *y*, *z*) positions of N, C, C^α^ and (where present) C^β^ atoms as functions of residue position along the sequence. Moving position restraints are then applied to the corresponding atoms to tug them smoothly towards their final target. Target positions are updated at every coordinate update (20 simulation timesteps), traversing one register unit every ten updates. The restraints remain active at their final target position until dismissed by the user *via* the widget, allowing the inspection and re­modelling of side chains prior to final release. In principle, in the absence of disulfide bonds and other branching modifications, any protein strand of arbitrary length and fold may be shifted in this manner.

The MCM2-7 hexamer contains five Cys_4_ zinc-finger domains. Although discussed in the original manuscript, the deposited structure did not include the coordinated zinc ions, and the most weakly resolved zinc finger (in chain 6) was presented with residues truncated to polyalanine and the cysteines well away from their canonical positions (Fig. 3 ▸ *c*). With improved connectivity in the map provided by *phenix.auto_sharpen* combined with the methods described above (in particular the extensive use of temporary position restraints to maintain tentative configurations while remodelling adjacent residues) I was able to ‘hand-fold’ the solution shown in Fig. 3 ▸(*d*), with neither clashes nor Ramachandran outliers and with the four cysteines coordinated around a zinc ion placed on the highest density local peak. Note: since metal-ion interactions in implicit-solvent MD do not adequately represent real-world behaviour, it was necessary to maintain permanent position restraints on the zinc ion and the surrounding S atoms for each zinc finger. In future versions of *ISOLDE* I plan to add metal-coordination bond/angle restraints to improve the handling of such situations. The need for improved handling of metal interactions is not unique to MD, as has been recently discussed in the context of crystallographic refinement of zinc-coordination sites (Touw *et al.*, 2016 ▸).

### 4.4. Finer-grained corrections   

Once satisfied with the overall gross fold of the model, the remainder of the task is pre­dominantly a matter of working through the list of outliers. Each point on *ISOLDE*’s Ramachandran plot (Fig. 2 ▸), when clicked, will select and focus on the associated residue, allowing a workflow much the same as working through the list of *cis*/twisted peptide bonds. While this task may seem daunting in such a large structure, it is somewhat reassuring (if not particularly surprising) to note that outliers tend to cluster together in space, such that starting a simulation centred on a given outlier will often lead to the correction of multiple others.

On a workstation with sufficient performance it may be preferable to instead perform a series of interactive simulations covering selections of a few hundred residues at a time, systematically correcting any errors identified and highlighted by the real-time validation. This approach can be more efficient in that it largely does away with the need to directly view the Ramachandran plot, and additionally reduces the overhead involved in repeatedly creating and destroying simulations. To work through the 3ja8 model once would require approximately 20–40 such simulations.

Once the number of Ramachandran outliers has been reduced to the order of 0.1%, it is advisable to again equilibrate the entire model against the map at some nonzero temperature (the default is 100 K). It is common to see a number of new outliers appear as formerly marginal residues are pushed into disallowed conformations by their changed surroundings. After two iterations of this approach, noting that the proportion of residues in favoured Ramachandran space remained below 90%, I saved the coordinates and [after adding the missing ADP residues and the lysine described in Fig. 3 ▸(*b*) using *Coot*] refined the model using *phenix.real_space_refine* using its default settings (including the use of Ramachandran and rotamer restraints). This improved the proportion of favoured residues to 92% at the expense of a slight increase in the number of outliers (0.43%) and the introduction of a small number of twisted peptide bonds. After re-equilibrating the result in *ISOLDE* I worked through the remaining Ramachandran outliers as well as the lowest-probability marginal residues. Re-equilibration at 100 K followed by settling at 0 K left a single *trans* proline outlier on the very edge of the α-helical region, which I was unable to resolve in the *ISOLDE* environment. After running a final round of *phenix.real_space_refine* without the use of Ramachandran restraints and using the input coordinates as a reference model for backbone torsions, I obtained a final result with the statistics described in Fig. 2 ▸(*c*), with an overall *MolProbity* score of 1.44.

### 4.5. Analysis of changes   

**Figure 4:**
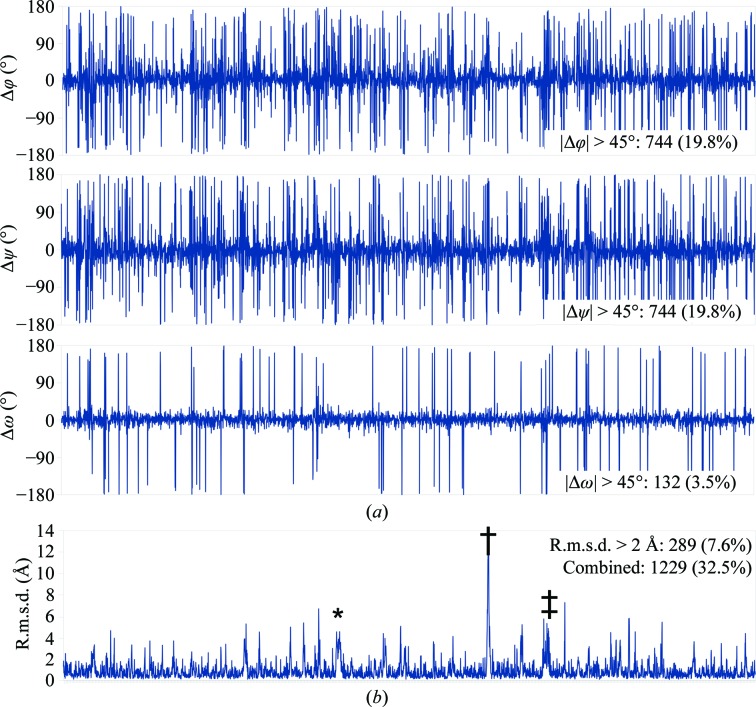
Per-residue changes in (*a*) backbone torsion angles and (*b*) heavy-atom positions highlight the fact that modest improvements in validation statistics may involve extensive changes to the model. Insets denote the numbers of residues with large changes in each criterion (defined as >45° change in dihedral angle or >2 Å heavy-atom r.m.s.d.). ‘Combined’ gives the number of residues with a large change in at least one criterion. Annotations in (*b*) refer to a single-residue register shift in chain 4, residues 469–497 (asterisk), a poorly interpretable loop originally modelled into density complicated by the unmodelled C-terminal end of chain 6 (dagger) and the zinc-finger domain of chain 6, which was a polyalanine trace in the original model (double dagger).

Looking only at the most commonly used overall validation statistics, the nonspecialist reader may easily be led to the conclusion that the changes made are quite modest. The clashscore (defined as the number of steric clashes ≥0.4 Å per 1000 atoms) has reduced from 32 to 2.35, the number of Ramachandran outliers has reduced from 1.15% to zero and the number of side-chain outliers has decreased from 0.1 to 0.03%. However, a more detailed residue-by-residue in­spection (Fig. 4 ▸) reveals that obtaining a stable model with these statistics required extensive changes throughout the structure. Almost 20% of all φ and ψ dihedrals (and 3.5% of all ω dihedrals) changed by more than 45°, while 7.6% of all residues moved by more than 2 Å on average. Overall, almost a third of all residues in the model met at least one of the above criteria. Perhaps most concerningly, re-refining the deposited coordinates with the most recent available version of *phenix.real_space_refine* with default parameters improved the *MolProbity* score from 2.51 to 2.09 (most notably reducing the clashscore to below 10), yet comparison of this with my final structure yielded comparable results to those described above.

**Figure 5:**
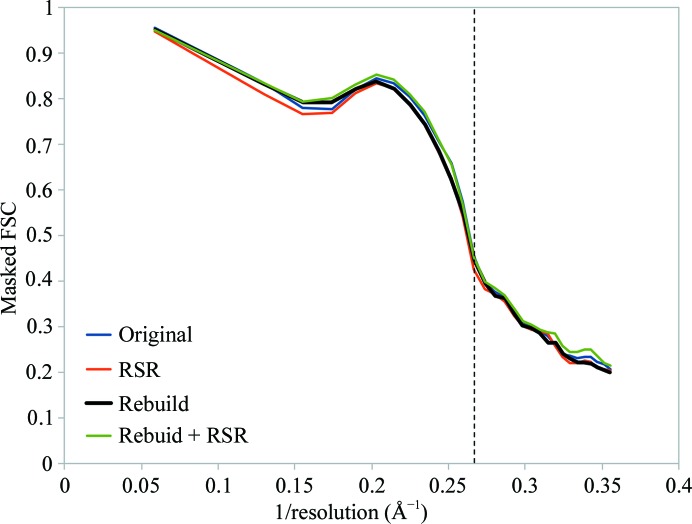
Extensive rebuilding yields minimal changes in correlation to the map. Starting from the original model (blue), real-space refinement with the current version of *phenix.real_space_refine* reduced the overall masked FSC from 0.828 to 0.816, with some improvement in geometry. Rebuilding in *ISOLDE* followed by a tightly restrained *phenix.real_space_refine* run as described (black) increased the FSC to 0.821, while running *phenix.real_space_refine* with default settings on the result (green) further increased the FSC to 0.830 with a slight degradation in geometry. These changes are far smaller than those arising from choice of map sharpening or inclusion/exclusion of H atoms, for example. The vertical dotted line denotes the published resolution of the map.

**Figure 6:**
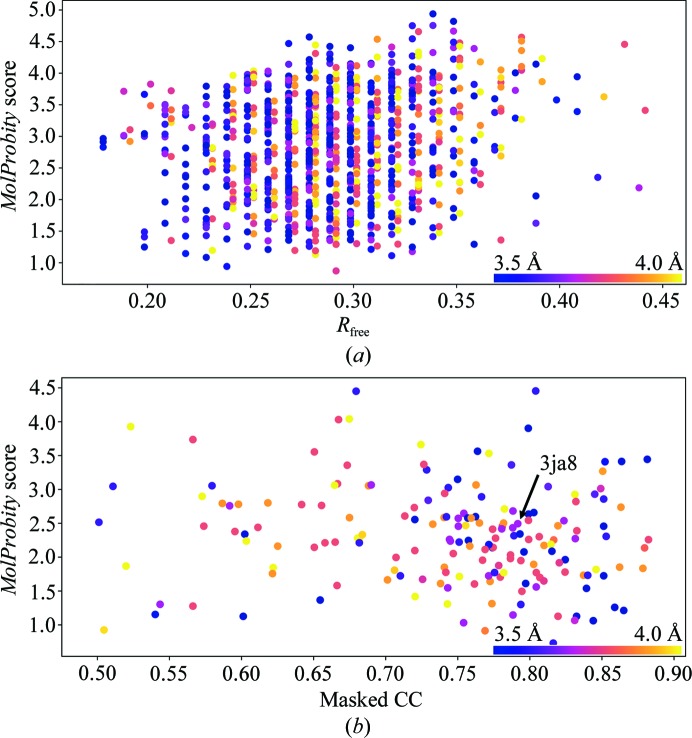
The relationship between model quality and fit to data breaks down in deposited 3.5–4 Å resolution crystal (*a*) and cryo-EM (*b*) models. The crystal structure cohort was limited to models deposited from 2007 to the present, and *R*_free_ values for data at ≤3.75 Å and >3.75 Å resolution are offset by ±0.0015 for clarity. All EM models in the resolution range with masked CC ≥ 0.5 were included in the analysis.

In light of the above, it is interesting to note that all changes to the model described here yielded minimal changes to the map–model Fourier shell correlation (FSC) as calculated using *phenix.model_vs_map* (Fig. 5 ▸). This prompted me to perform a brief analysis, comparing the *MolProbity* score with the fit to data for all 3.5–4.0 Å resolution crystal structures deposited in the last decade and all 3.5–4.0 Å resolution cryo-EM models deposited to date (Fig. 6 ▸). All statistics other than EM model–map correlation were gathered from the wwPDB validation reports (Read *et al.*, 2011 ▸); the latter were gathered from a previous analysis (Afonine *et al.*, 2018 ▸). The *MolProbity* score was calculated from clashscore, rotamer outliers and non­favoured Ramachandran statistics as described previously (Chen *et al.*, 2010 ▸).

While it is indisputable that at high resolution model geometric quality closely correlates with the fit to data, it is clear that at these low resolutions this relationship has broken down. This is perhaps not entirely surprising; after all, the fact that many unphysical models may fit a low-resolution map is the very reason restraints are necessary in the first place! However, this does emphasize the ever-increasing need for more restraints at low resolution, and in particular indicates that the choice to accept a loss of geometric quality in return for a slight gain in fit to the density is one that must be made with great care.

## 5. Discussion   

### 5.1. With great prestige comes great responsibility   

Being the first to solve and publish the structure of an important biological complex is a huge achievement which rightly brings with it substantial prestige. The structure discussed here is no exception: it is clearly the result of extensive painstaking experimental and computational work, deserving (in my opinion) of its publication in *Nature*. However, when one is the first to publish a structure the responsibility is greater than at any other time to ensure that the structure is as correct as possible, since (at least until substantially higher resolution data are collected) it is almost certain to become the template upon which future studies of the same complex are based. This case is no exception: since the deposition of PDB entry 3ja8 in 2015 a further ten MCM-2 structures have been published by various authors, all of which display a cohort of *cis* and twisted peptide bonds (mean 121, minimum 87, maximum 140) which overlap substantially with the original structure (Supplementary Fig. S4). As the pace of structural biology depositions grows ever faster, this propagation of errors through the database seems likely to become more problematic.

### 5.2. Advantages and drawbacks of MD methods   

The need for more and more prior information (in the form of restraints) as the resolution of a data set decreases is a well established truism in structural biology. In this context, MD methods may be thought of as adding a very large yet difficult to quantify set of additional restraints. Where traditional model-building tools may consider only bonded interactions and refinement tools aim to reduce nearest-neighbour atomic clashes, in a typical MD simulation each individual atom ‘feels’ van der Waals and electrostatic force contributions from all atoms within a 10 Å radius: on the order of 1000 individual pairwise forces for an atom in a well packed hydrophobic core. Provided that the parameterization of these interactions is a reasonable facsimile of reality, it is unsurprising that their inclusion leads to improved results.

In particular, it can be argued that the clashscore is the most sensitive of all of the standard conformational validation metrics: whereas most Ramachandran and rotamer outliers represent unusual strained conformations, a substantial overlap of nonbonded atoms is a physical impossibility under any conditions conducive to known life. Models with good Ramachandran and rotamer statistics but poor clashscore should therefore be treated with particular caution. I would argue that this caution should extend to low-resolution models with large numbers of missing side chains, since removing the side chain gives the ability to achieve ‘allowed’ backbone conformations that would cause severe clashes if the side-chain atoms were present. The impossibility of clashes is strictly reflected in the MD environment, with van der Waals energy rising to infinity with 1/*r* ^12^ for small interatomic distances. Every step of an MD simulation therefore represents a structure with a clashscore very close to zero, which combined with the strict inclusion of complete residues and the explicit treatment of electrostatics leaves conformational errors with fewer places to ‘hide’ compared with traditional approaches.

A corollary to the above is that problems will arise where classical MD parameterization is insufficient to replicate the real-world behaviour of some chemical species. A well established example is metal ions (Rode *et al.*, 2005 ▸), where substantial charge transfer (beyond the scope of classical MD) often occurs between the ion and its neighbours. While reasonable agreement with experiment is achievable with careful parameterization in explicit solvent (Li, Song *et al.*, 2015 ▸), naive inclusion of these ions in an implicit solvent environment leads to severe overestimation of long-range electrostatic interactions, causing unmanageable distortion of the surroundings. Given the many significant challenges facing the use of explicit solvent in a model-building environment, it appears prudent to carefully consider the best path forward here. As a short-term (yet rather unsatisfying) measure, simply artificially setting all metal-ion charges to +1.0 alleviates the overestimation of long-range electrostatics while maintaining reasonable van der Waals radii, allowing their use with the careful application of suitable restraints with the aid of the experimental map (as used here for handling the zinc-finger domains). A near-term goal in *ISOLDE* is the addition of automatically applied, user-adjustable distance and angle restraints for metal centres. In the medium term, it may prove valuable to develop a library of building blocks consisting of metal–water clusters parameterized with varying levels of completion of the first hydration shell. In the longer term, it may be practical to provide an *ab initio* quantum-mechanical treatment of these and other unusual sites, although it is likely to be some time before single-workstation interactive performance is possible.

It also must be noted that classical MD parameterizations do not explicitly maintain the chirality of any given centre. Rather, changes in chirality are blocked by energy barriers that are effectively insurmountable under normal equilibrium conditions. Chirality flips may occur under extreme conditions, however; for example, in the vicinity of a complex multi-atom clash in the starting model. While such events should be very rare, I plan to add explicit chirality restraints in a future version of *ISOLDE*. In the meantime, the chirality of all centres in the final model should be checked with a tool such as *MolProbity*.

## 6. Conclusions and future directions   

For the purposes of this manuscript, I have demonstrated that the *ISOLDE* environment combined with an existing refinement package allows a single user, working on a moderately priced workstation, to rebuild a large, low-resolution structure to near-atomic resolution standards in approximately one week of work, without reference to external information such as reference models. This is not intended as a suggestion that such extensive manual interaction is necessary or desirable. In fact, it is likely that a majority of the improvements identified, in particular those that involve simply flipping 1–2 adjacent peptide bonds, should be readily manageable by automated methods such as those recently described for use in moderate-resolution crystal structures (Touw *et al.*, 2015 ▸). In addition to the many possible permutations in the use of *ISOLDE* with external tools in a larger workflow, there is substantial scope for the automation of various common tasks (and the implementation of existing successful algorithms) using combinations of the various unit operations defined in *ISOLDE* itself. A simple example of such a combination is the semi-automated shifting of protein residues in register described in §[Sec sec4.3]4.3, which is accomplished by the concerted action of many moving position restraints.

Detailed inspection of the changes made to this model as summarized in Fig. 4 ▸ presents some cause for concern, in particular when contrasted against the minimal change in correlation to the map (Fig. 5 ▸). While the number of outliers by any standard validation metric changed by an apparently modest amount (no more than 3.2% of residues/atoms) in reality almost one third of all residues underwent a marked change in backbone conformation and/or position. That the conformation of the model can vary so substantially with such modest changes in the resulting statistics strongly supports the use of MD and similar environments, since these help to exclude many configurations which are statistically ‘allowed’ but energetically unfavourable.

## Supplementary Material

Supplementary Data.. DOI: 10.1107/S2059798318002425/ic5101sup1.pdf

## Supporting information

Moving Picture Experts Group (MP4) video file https://doi.org/10.1107/S2059798318002425/ic5101sup2.mp4. Extended video demonstration of ISOLDE.
