# Supplementary material for: ISOLDE: a physically realistic environment for model building into low-resolution electron-density maps
Source: Acta Crystallogr D Struct Biol. 2018 Apr 11;74(Pt 6):519–30. doi: 10.1107/S2059798318002425 (PMC6096486; doi:10.1107/S2059798318002425)
Supplement: Supplementary file 1 [file d-74-00519-sup1.pdf]

STRUCTURAL BIOLOGY

Volume 74 (2018)

Supporting information for article:

ISOLDE: a physically-realistic environment for model building into low-resolution electron-density maps

Tristan Ian Croll

S1. Relationship between model size and resolution

Resolution and molecular weight data for all crystallographic models deposited as of 07 November 2017 were recovered from the RCSB PDB server (www.rcsb.org).

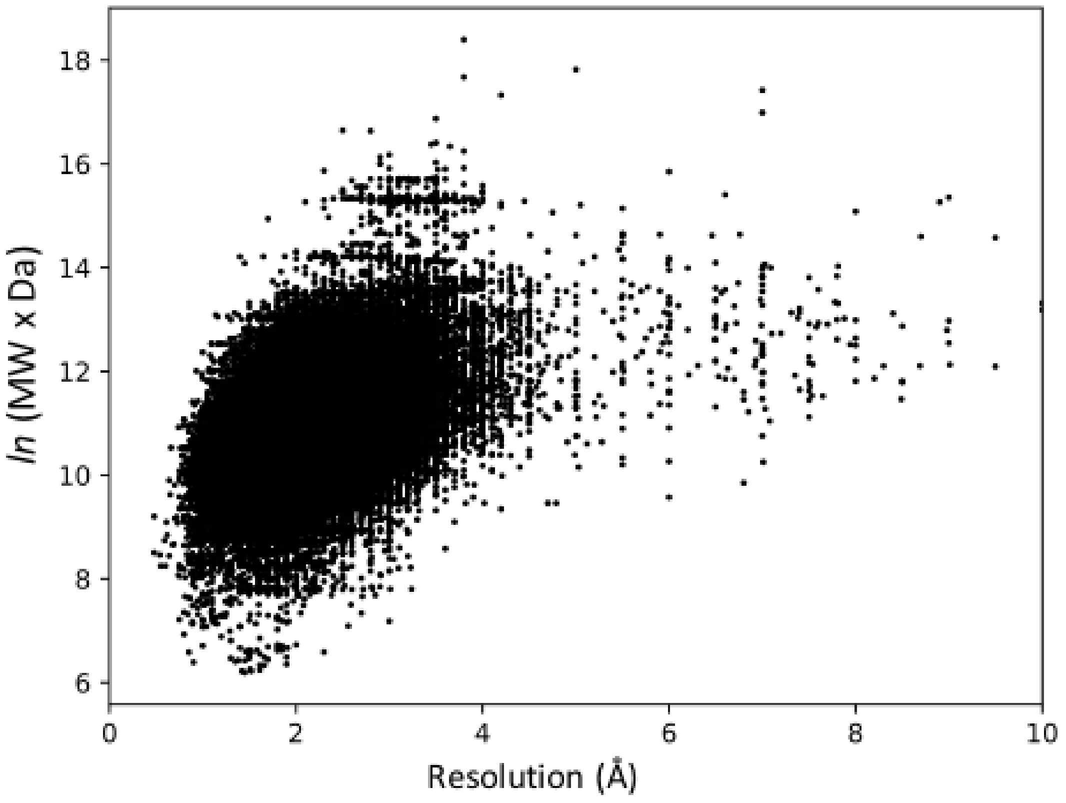

Figure S1 Low-resolution crystal structures tend to be larger than high-resolution ones. The mean 3.5-4Å asymmetric unit (608 kDa) is approximately 11.5 times larger than the mean of the ≤2Å cohort (53 kDa).

S2. Masking of maps to an atomic selection

Selecting one or more atoms in the main *ChimeraX* window and clicking “Mask map(s) to selection” in *ISOLDE* results in a visualisation similar to that in Figure S2, with all maps masked to within 5Å of the selection. While this will work for any arbitrary atom selection, in practice it is most useful for isolating continuous sequences or *β*-sheets for examination. This is performed automatically every time a new simulation is started.

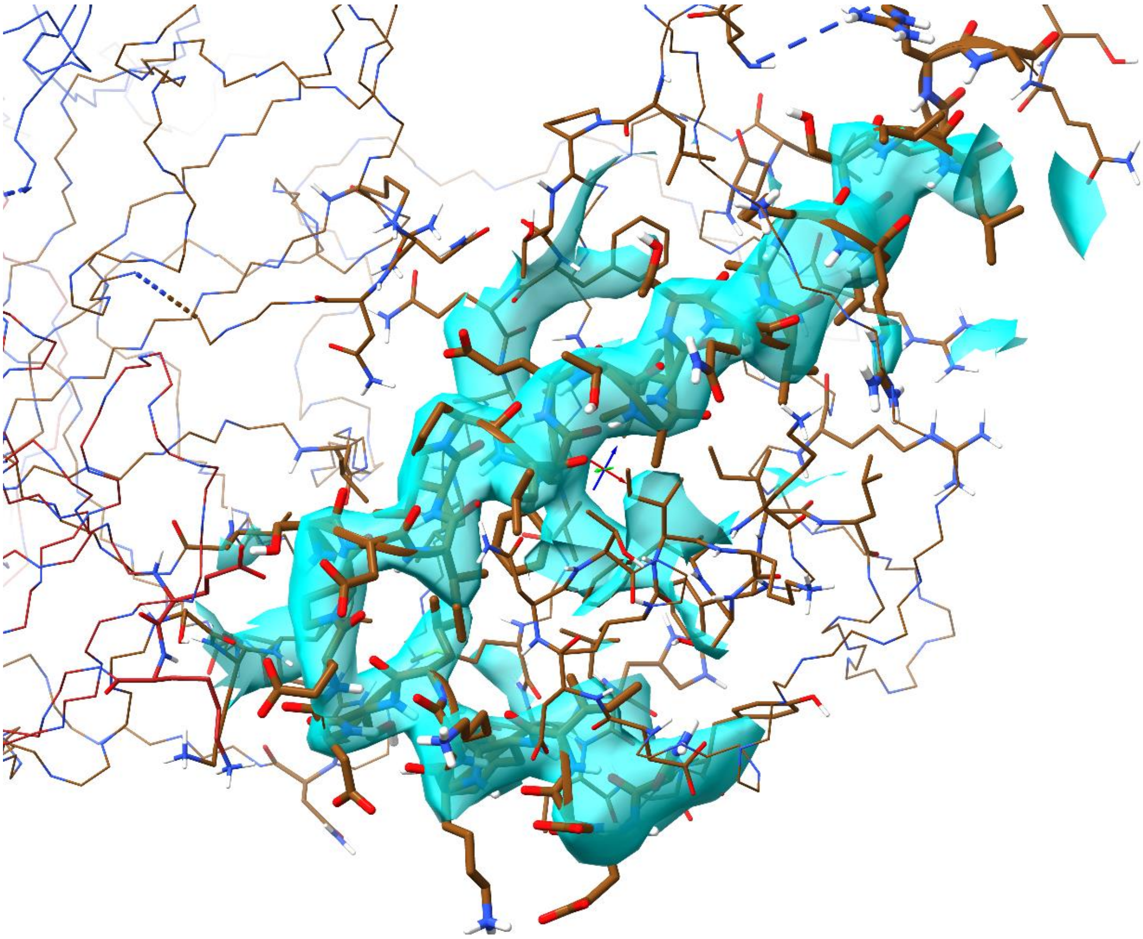

Figure S2 Interactive map masking eases interpretation of complex regions. Here a helix-turn-helix (thicker sticks and ribbon) has been selected for inspection. Immediately surrounding residues are shown in a thinner stick representation, while the remainder of the structure is reduced to a minimal backbone trace.

S3. Performance benchmarks

Benchmarks were run using identical selections encompassing part or all of the MCM-2 complex on the two recent-model laptop computers ("ultrabook" and "desktop replacement") described in Table S1. The equilibrated MCM-2 model was loaded in ISOLDE along with the sharpened map, and simulations were started from identical selections on each machine. The desktop replacement machine supported usable interactive simulations up to approximately 20,000 atoms (approx. 1,200 protein residues). The ultrabook, meanwhile, was able to run marginally interactive simulations (3-5 coordinate updates per second) up to approximately 4,000 atoms, sufficient for remodelling around e.g. individual residues or loops. Simulation of the entire 60k atom MCM-2 complex is possible on the ultrabook – while the speed is far too low to be interactive, it would be sufficient to non-interactively settle the model into the map within 10-20 minutes.

Table S1 Specifications of computers used for benchmarking.

	Ultrabook	Desktop replacement
Make/Model	Apple MacBook Air (13 inch, early 2015)	Asus ROG Strix GL502V
CPU	Intel Core i5, 1.6 GHz	Intel Core i7 6700HQ, 2.6GHz
GPU	Intel HD Graphics 6000	NVIDIA GTX 1070
RAM	8GB	32GB
GPU RAM	1536MB	8GB
OpenCL/CUDA cores	48	1920

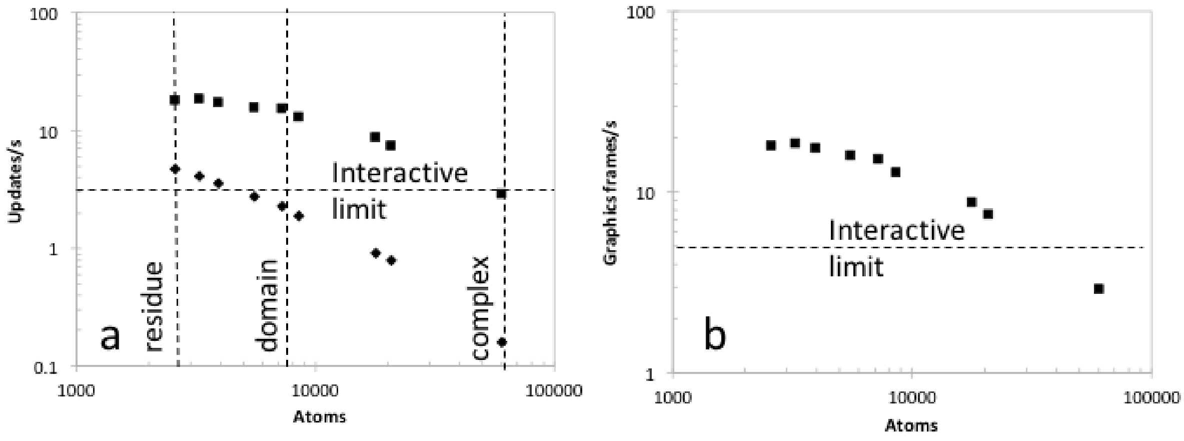

Figure S3 (a) Benchmark performance of the ultrabook (diamonds) and desktop replacement (squares) for different simulation sizes. Vertical lines illustrate typical simulation sizes resulting from selection of an individual residue, a moderately-sized (~100-residue) domain, or the entire complex. Speeds less than approximately 3 updates per second (horizontal line) represent the lower limit of usability for interactive modelling. Each coordinate update is the result of 20 simulation steps. (b) The MacOS GPU driver implementation prioritises graphics over OpenCL performance, so rendering continues at 30-60 frames per second (fps) regardless of simulation size. In Linux OpenCL and graphics have equal priority, so the frame rate drops with increasing simulation size. Nevertheless, usable frame rates are maintained for simulations up to approximately 20,000 atoms.

S4. With great prestige comes great responsibility

For all eleven current structures containing the MCM-2 complex (3ja8, 3jc5, 3jc6, 3jc7, 5bk4, 5h7i, 5u8s, 5u8t 5udb, 5v8f, 5xf8) I extracted the chains common to 3ja8 and analysed them using *phenix.molprobity*. For each peptide bond that appeared in a *cis* or twisted conformation in any structure, I counted the number of times the same bond was *cis* or twisted in any of the other structures (0). While this analysis is hampered slightly by varying levels of completeness of the different structures, it is clear that the majority of erroneous peptide bonds in 3ja8 have carried through to subsequent models.

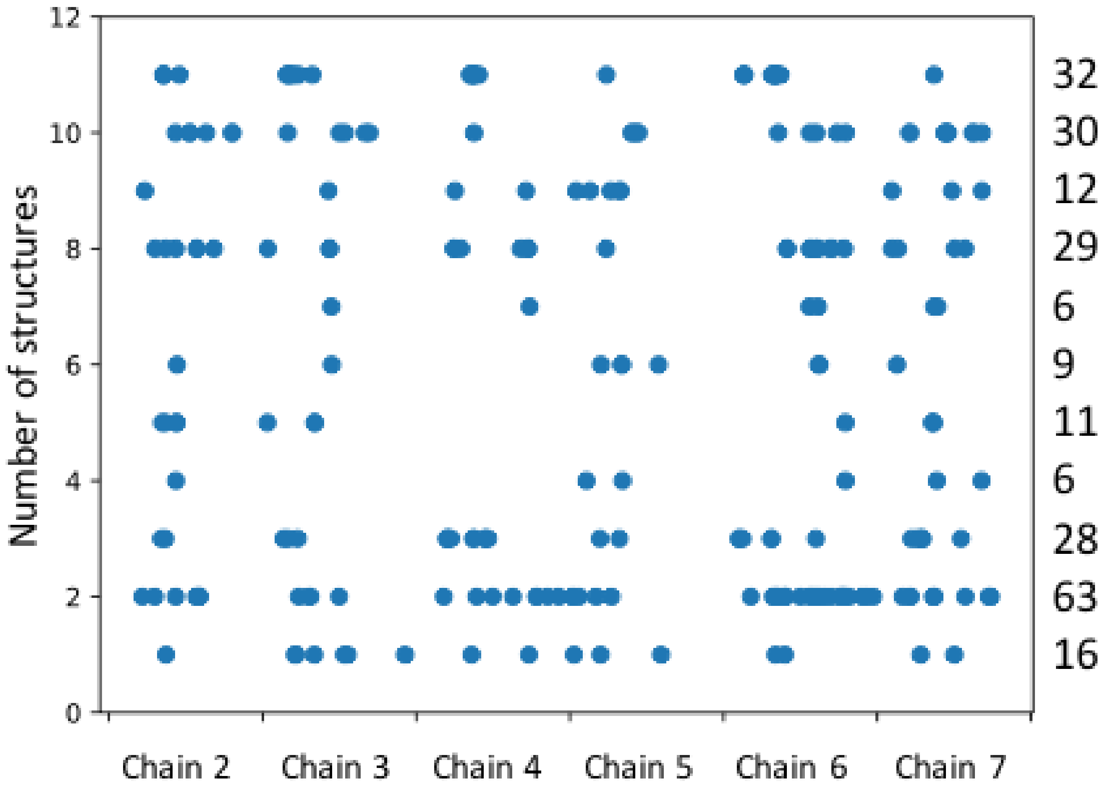

Number of times each residue appears in *cis* or twisted conformation amongst the eleven current structures of the MCM-2 complex. Each number on the right-hand side gives the count of *cis* points in the corresponding row. Many errors appearing in the original 3ja8 have been carried through to all subsequent models, and only 16 such conformational errors are unique to a single structure
